# The Devastating Effects of Sleep Deprivation on Memory: Lessons from Rodent Models

**DOI:** 10.3390/clockssleep5020022

**Published:** 2023-05-15

**Authors:** Pinqiu Chen, Weikang Ban, Wenyan Wang, Yuyang You, Zhihong Yang

**Affiliations:** 1 Institute of Medicinal Plant Development, Chinese Academy of Medical Sciences and Peking Union Medical College, Beijing 100193, China; 15695197298@163.com (P.C.);; 2 Key Laboratory of Molecular Pharmacology and Drug Evaluation, Ministry of Education, School of Pharmacy, Yantai University, Yantai 264005, China; 3 School of Automation, Beijing Institute of Technology, Beijing 100081, China

**Keywords:** sleep deprivation, memory impairment, hippocampus, mechanism, progress

## Abstract

In this narrative review article, we discuss the role of sleep deprivation (SD) in memory processing in rodent models. Numerous studies have examined the effects of SD on memory, with the majority showing that sleep disorders negatively affect memory. Currently, a consensus has not been established on which damage mechanism is the most appropriate. This critical issue in the neuroscience of sleep remains largely unknown. This review article aims to elucidate the mechanisms that underlie the damaging effects of SD on memory. It also proposes a scientific solution that might explain some findings. We have chosen to summarize literature that is both representative and comprehensive, as well as innovative in its approach. We examined the effects of SD on memory, including synaptic plasticity, neuritis, oxidative stress, and neurotransmitters. Results provide valuable insights into the mechanisms by which SD impairs memory function.

## 1. Introduction

**Figure 1:**
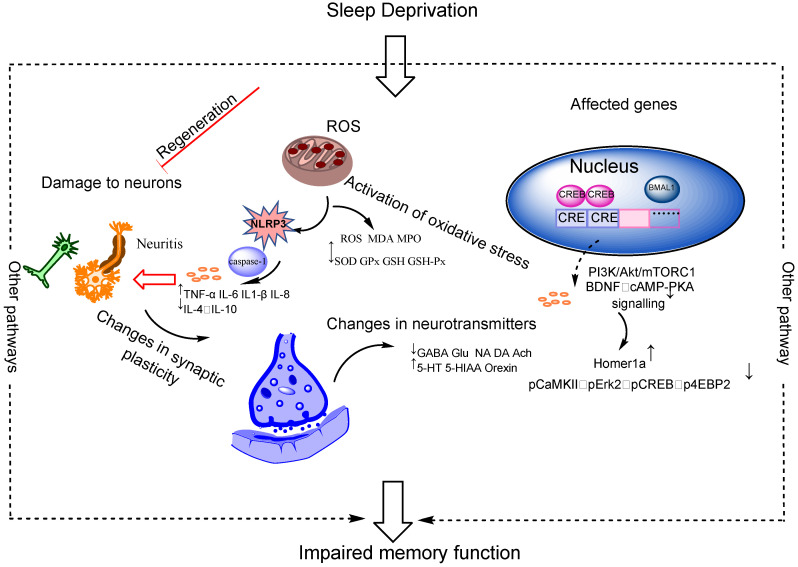
Potential mechanisms for the effects of partial sleep deprivation on memory function. The effects of SD on memory function in terms of synaptic plasticity, neuroinflammation, oxidative stress, genes, and neurotransmitters. Abbreviations: TNF-α (tumor necrosis factor alpha); IL-6 (interleukin-6); IL-1β (interleukin-1β); IL-8 (interleukin-8); IL-4 (interleukin-4); IL-10 (interleukin-10); ROS (reactive oxygen species); MDA (malondialdehyde); MPO (myeloperoxidase); SOD (superoxide dismutase); GPx (glutathione peroxidase); GSH-Px (glutathione peroxidase); GABA (gamma-aminobutyric acid); Glu (glutamic acid); NA (norepinephrine); DA (dopamine); Ach (acetylcholine); 5-HT (5-hydroxytryptamine); 5-HIAA (5-hydroxyindoleacetic acid); BDNF (brain-derived neurotrophic factor); ↓: fall;↑: rise.

Various factors contribute to SD in the modern world, and these include alcohol consumption, shifting work, exposure to excessive light and noise, stress, anxiety, and certain medical conditions. Insomnia, narcolepsy, restless leg syndrome, and obstructive sleep apnea are some of the manifestations of SD. It is experienced by many people to varying degrees. More than one-third of the global population suffers from insomnia, with prevalence rates ranging from 23.2% to 27.1% [1]. Additionally, the insomnia rate has increased to 29.7–58.4% during the epidemic [2]. Overall, the prevalence is higher in Asian and European regions, especially in East Asia, where it is about 27% [1,3]. In China, about 38% of the population suffers from various types of SD, and this rate is higher than the world average of 27%. Nearly half of older adults aged more than 60 years have experienced SD. More than a third of American adults have less than 6 h (h) of sleep per night. People with SD are more likely to develop diabetes, high blood pressure, heart disease, chronic kidney disease, and Alzheimer’s disease by up to two to three times more than those without SD [4]. Encoding, consolidation, and retrieval are the three stages of memory formation [5,6]. During sleep, memory functions, including spatial memory, memory recognition, long-term memory, short-term memory, and prospective memory, are maintained and strengthened. Several studies suggest that SD can decrease hippocampal activation during the encoding phase while in the awake period, resulting in impaired memory retrieval even after one night of recovery sleep [7]. Total SD negatively affects their ability to form trace-conditioned memories [8]. Memory has been shown to be highly affected by SD. As a result of SD, patients have varying degrees of impairment in their prospective and spatial memory [9,10,11,12,13]. Memory impairment due to SD is a hot and difficult issue in current research. SD can result in memory impairment through a variety of mechanisms, but few studies have attempted to systematically and purposefully summarize the underlying mechanisms. Therefore, clarifying the substantive effect of SD on memory is extremely critical. This article aims to provide an overview of the relationship between sleep and memory function, as well as the mechanism of memory impairment caused by SD. A few studies reported that SD may improve memory, but others stated that SD increases the risk of various diseases and negatively affects learning and memory [14,15,16,17]. We selected 21 representative articles from all the references cited in the review that were highly relevant to the topic, and summarized them to provide a comprehensive overview of the existing literature on the specific topic. Our focus in this article is on the detrimental effects of SD on memory (Figure 1).

### Method 

This is not an exhaustive or systematic review. The aim of this literature review is to explore the effects of SD on memory function and to advance the field by describing relevant mechanisms. Electronic searches were conducted in the PubMed, Google Scholar, Web of Science, and CNKI databases for recent publications, using the following search terms: Sleep Deprivation AND Sleep; Memory Function AND Sleep; Insomnia AND Memory Function; Sleep Deprivation and Memory Impairment; Sleep Deprivation AND Mechanisms of Injury. Initially, we focused on recent publications and subsequently reviewed the reference sections of relevant articles to identify older publications that aligned with our research goals. Finally, we selected the most recent articles that provided current and relevant information on our predefined subtopics.

The present review is structured into the following sections: (a) Impaired Memory Function, (b) Potentially Relevant Mechanisms (Synaptic Plasticity, Neurons, Oxidative Stress, Genes, Neurotransmitters, Circadian Rhythms, Rodent-to-Human Complexities), and (c) Summary and Prospects.

## 2. Impaired Memory Function

**Figure 2:**
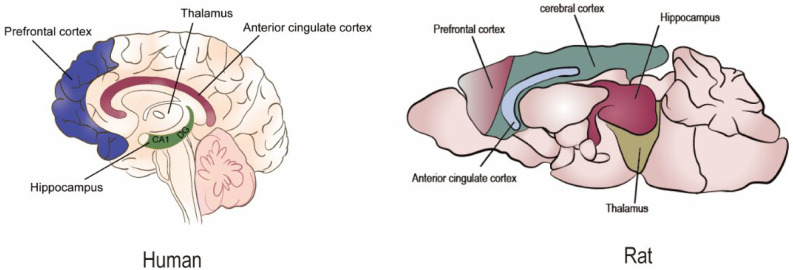
The main brain areas affected by sleep deprivation in humans and rodents.

Insomnia is the most prevalent type of sleep disorder and is characterized by a high rate of morbidity and mortality, as well as high social costs. A growing body of evidence suggests that sleep plays a critical role in memory because it is a normal physiological process and a crucial brain function [18]. Alzheimer’s disease, Parkinson’s disease, and dementia are all associated with SD. Several areas of the brain damaged by SD include the hippocampus, thalamus, prefrontal cortex, and anterior cingulate cortex (Figure 2). The hippocampus is responsible for the processing, consolidation, and retrieval of short- and long-term memory and for spatial navigation and orientation [6,19]. Proteins that are linked to the hippocampus are crucially involved in memory formation [20]. SD could induce damages to hippocampal neurons and reduce the size and volume of the hippocampus, impairing hippocampal-dependent memory functions and resulting in difficulties in recalling past events and forming new memories [21]. SD can affect hippocampal function at the molecular level by decreasing encoding-related activity within the hippocampus [22]. The thalamus is involved in regulating sleep–wake cycles during SD and emotional processing [23]. Similarly, SD can lead to decreased activity in the thalamus, resulting in impaired sensory perception and processing [24]. The encoding of working memory relies on the prefrontal cortex, which plays a crucial role in attention and memory [25,26]. Reduced prefrontal cortex activity caused by SD can impair cognitive function, including attention, working memory, and fear memory consolidation [27,28]. The anterior cingulate cortex modulates the frontoparietal functional connectivity between resting-state and working memory tasks [29]. Additionally, SD can cause decreased anterior cingulate cortex activity, leading to impaired emotional regulation and decision-making abilities [30,31]. Insomnia is often associated with a decline in memory function, indicating a strong connection between insomnia and memory loss.

SD has been reported to negatively affect memory in animal models and humans [32,33]. It can impair spatial working memory in humans [34], as well as in young rats after 24 h of SD [35]. SD with 4–6 weeks of chronic rapid eye movement sleep (REM) can affect hippocampal spatial memory, short-term memory, and long-term memory in Wister rats [36,37,38]. The induction of short- and long-term memory in *Aplysia* was inhibited by 9 h/12 h SD [39]. The short- and long-term memory was significantly impaired by SD in rats and sea rabbits [40,41]. Additionally, prospective memory and declarative memory are also affected by SD [13,42], resulting in attenuation of weakly encoded memories in humans [43] and impaired encoding of trace memory in rats [8]. Although SD has a great effect on the memory function of older individuals [44], healthy young men experienced impaired working memory after 16 h of total SD [45]. After total SD with multiplatform and mild stimulation, EPM-M1 male mice developed memory deficits in the plus-maze discriminative avoidance task and passive avoidance dance task. REM SD for 72 h impaired novelty-related object site memory in mice [46]. In a study using C57BL/6J mice, a mild stimulus method of SD was used to train them for 1 h on a location recognition task, which significantly impaired memory function after only 3 h [47]. When flies were exposed to 4 h of SD during the consolidation phase, their memory was disrupted [48]. Memory function is also impaired in *Octodon degus* [49] and zebrafish [50]. Multiple animal models demonstrated the effects of SD on memory. Memory processing is severely affected by acute or chronic SD (Table 1).

## 3. Potentially Relevant Mechanisms

### 3.1. Disruption of Synaptic Plasticity

In the hippocampal nervous system, synaptic plasticity serves as the neurobiological basis for learning and memory. Multiple studies have demonstrated that sleep enhances synaptic plasticity, promotes hippocampal neuron survival, and facilitates the consolidation of memory. SD, however, impairs learning and memory by disrupting hippocampal function and plasticity [61,62]. In addition to altering synaptic plasticity in different hippocampal regions (DG and CA1), SD inhibits cell proliferation in the dentate gyrus region of the hippocampus, resulting in neurological impairments in the hippocampus and ultimately reduced learning and memory function [63,64,65,66,67]. According to one study, SD for 5 h specifically decreased dendritic spines in the CA1 area of the hippocampus while increasing the activity of cofilamentous actin. This memory-damaging effect was found to be associated with the activation of the cAMP-PDE4-PKA-LIMK-cofilin signaling pathway [68].

Alterations in brain-derived neurotrophic factor (BDNF) may contribute to memory impairment. BDNF promotes neuronal and synaptic growth and differentiation to regulate synaptic plasticity. When BDNF levels are reduced, neuroplasticity may be disrupted, which is essential for memory function [69,70,71,72,73]. In the rodent hippocampus, BDNF induces persistent synaptic strengthening by activating the MAPK/Erk pathway. Sleep disorders may be associated with reduced levels of BDNF according to meta-analyses [74]. Insomnia may be affiliated with reduced BDNF levels [75]. Sleep-deprived college students were reported to have lower plasma BDNF levels than normal students [76]. In the hippocampus, SD alters BDNF/TrkB/Erk signaling and reduces synaptic efficacy. In chronic and total SD, serum BDNF levels decrease and memory function is compromised [77,78]. In rats exposed to RSD for 24 h, BDNF levels in the hippocampus and prefrontal cortex were reduced [79]. Furthermore, the BDNF protein and mRNA levels were significantly reduced in the hippocampus of Wistar ovariectomized rats after 72 h of SD [80]. SD also altered approximately 70% of relevant protein levels [81]. pCREB plays a crucial role in neuronal growth, survival, and memory formation as another indicator of synaptic plasticity [82]. Interestingly, SD for 3 h per day for 30 days decreased the spatial learning and memory abilities of mice and significantly decreased the protein expression level of pCREB in the hippocampus of mice [83]. As a result of changes in synaptic protein expression patterns, SD may influence memory function.

Synaptic transmission is characterized by long-temporal inhibition and long-temporal enhancement (LTP) [84]. The effects of SD on LTP induction in the hippocampus and synaptic time course enhancement have been reported [85,86]. The capacity of rat hippocampal slices to undergo LTP is compromised under conditions of SD [87]. After being subjected to 3 h of training followed by 1 h of SD, rats showed impaired hippocampal synaptic plasticity and memory function [47]. In an in vitro study, REM SD inhibits LTP. DG LTP induction was suppressed after 24 h of SD in rats. Cellular LTP in the DG region was found to decrease with time in rats after different periods of REN SD, with 3 h of SD reducing the LTP from 38.7% to 7.6%; hence, only 3 h of SD could result in changes in synaptic plasticity [82,88]. The single-platform method of SD at 24, 48, and 72 h was found to damage LTP in the hippocampal CA1 region, and the damage was more severe at 72 h than at 24 h [89,90]. Therefore, even a short duration of SD can lead to a significant reduction in synaptic plasticity, particularly in the DG region.

Impaired synaptic plasticity deepens with prolonged deprivation. However, SD enhanced LTD inhibition; after 12 h SD by mild treatment, the LTD of EPSP in the CA1 region of the rat hippocampus was enhanced by approximately 20%, resulting in a concurrent decrease in memory acquisition and consolidation [91].

### 3.2. Affecting Neurons

#### 3.2.1. Damage to Neurons of the Hippocampus

SD increases neuronal damages and induces the focalization of neuronal cells [92]. Upon activation, p38MAPK plays a negative regulatory role in the occurrence and development of central nervous system diseases. According to a study, prolonged sleep disruption inhibits the regeneration of hippocampal neurons and impairs memory. Moreover, sleep disruption disrupts astrocytes, activates p38MAPK, and contributes to neuronal injury [93,94]. A modified multi-platform method was used to subject rats to SD for 1, 3, 5, and 7 days; results showed that the memory capacity of the rats decreased as the SD duration increased. The HE staining revealed significant structural damage to hippocampal neurons, as well as an increase in phosphorylated p38MAPK-positive cells, suggesting that SD could activate p38MAPK in the hippocampal area and mediate the process of SD damage to neuronal cells [95]. Another study found that SD negatively affects memory function, which may be related to lower levels of the AKAP150 protein in the hippocampus and decreased AMPA receptor activity [96].

#### 3.2.2. Neurological Inflammation

A number of studies have shown that neuroinflammation contributes to impaired learning and memory functions [17,94,97]. When neuroglia are activated, pro-inflammatory cytokines are released in the brain, resulting in a decrease in cognitive function [98,99,100,101]. SD induces spatial memory deficits in rats by activating microglia and glial cells in the hippocampal region [57]. C57BL/6J male mice subjected to 1 and 7 d of 24 h/day SD had impaired hippocampal-dependent memory functions, activated microglia in the mouse hippocampus, and increased pro-inflammatory cytokine IL-6 levels, leading to hippocampal neuritis [17]. Chronic SD for 21 d for 20 h per day exacerbated impairment of recognition memory, working memory capacity, and conditioned fear memory capacity in 3xTg-AD mice. These lesional effects are closely associated with neuritis caused by excessive microglial activation [32]. Chronic SD promotes the transformation of microglia into the neurotoxic M1 phenotype and subsequently induces the NF-κB pathway to increase the release of pro-inflammatory factors (TNF-α, IL-6), causing neuroinflammatory response, aggravating hippocampal neuron damage, and affecting cognitive function [55]. Further studies found that SD increased the protein levels of the pro-inflammatory cytokines TNF-α, IL1-β, IL-6, and IL-8 but decreased the protein levels of the anti-inflammatory cytokines IL-4 and IL-10 in the rat hippocampus [55,57,102,103,104]. Serum levels of pro-inflammatory cytokines (IL-6, IL-1β, TNF-α) increased in SD rats after 24 h of SD, and the effect was more significant in cerebrospinal fluid after 72 h of SD (IL-1β: 30.2 ± 12.8 pg·mL^−1^→43.6.8 ± 9.4 pg·mL^−1^; IL-6: 28.8 ± 9.3 pg·mL^−1^→37.8 ± 7.4 pg·mL^−1^; TNF-α:19.1 ± 6.3 pg·mL^−1^→21.8 ± 7.4 pg·mL^−1^). Hence, SD could induce neuroinflammation by inducing microglial pro-inflammatory cytokines [105]. CSD can trigger low-grade neuroinflammation, leading to focal recognition memory impairment. In addition, SD increased the inflammatory cytokine (TNF-α, IL-1β) levels in rats, accompanied by the activation of NF-κB and AP1 transcription factors in the hippocampal and pear-shaped cortical regions [102]. NRP3 has an essential role in SD-induced neuroinflammation [51,92,106]. SD for 5 weeks promoted the activation of the NLRP3/cysteine aspartame 1 pathway in adult male C57BL/6 mice; this finding reveals that the P38 and ERK-MAPK signaling pathways are involved in SD-induced activation of the NLRP3/focal death axis [106]. These results suggest that SD may increase the levels of pro-inflammatory cytokines in brain tissue, particularly in the hippocampus.

Creating long-term memory requires a cellular program in neurons, astrocytes, and glial cells of the brain, which play a necessary role in this program by converting glycogen to lactate and transporting it to neurons [107,108,109]. Microglia are found to be crucial for protecting fear-conditioning memories formed during the recovery sleep after a period of SD [110]. SD-induced memory impairment by regulating microglial phagocytosis is accomplished by CD33/TREM2 signaling [111]. SD induces spatial memory impairment in rats through altered neuroinflammatory responses and glial cell activation in the hippocampus [57]. Further data presented a correlation between spatial memory impairment and activated microglia induced increased pro-inflammatory cytokines after 48 h of SD [57]. Inhibiting the microglia activation improves the spatial memory and adult neurogenesis in rat hippocampus during 48 h of SD [112].

### 3.3. Activation of Oxidative Stress

Several studies have linked SD-induced memory impairment to increased oxidative stress within the hippocampus [55,63,113,114]. Memory formation is critically affected by oxidative stress [115]. The increased oxidative stress caused by CSD impairs short-term and long-term memory [116,117,118]. CSD also downregulates BMAL1 expression and induces excessive oxidative stress [55]. SD significantly increased the ROS, MDA, and MPO levels in the hippocampus of mice and decreased the activities of SOD, GPx, GSH, and GSH-Px enzymes. SD could be responsible for abnormal metabolism of hippocampal tissue, enhanced lipid oxidation, and lesioned hippocampal tissue [95,119]. Moreover, SD significantly increased the levels of TBARS and GSSG and decreased the GSH/GSSG ratio and the catalase activity, suggesting that SD could reduce antioxidant mechanisms and impair short- and long-term memory [114]. Similarly, SD was observed to affect learning and memory in young rats due to decreased expression levels of neuronal nitric oxide synthase (n NOS) in the prefrontal cortex and hippocampus [120]. Furthermore, during acute SD, TBARS or lipid peroxidation levels were elevated in the hippocampus, hypothalamus, thalamus, and cortex [121,122]. In another study, rats subjected to various forms of intermittent SD showed impaired memory and learning abilities, especially when the duration of the SD episodes was longer and the intervals between the SD episodes were shorter. SD also inhibited the SOD activity in the cerebral cortex, hippocampus, and plasma and increased the MAD content and TChE activity [123].

### 3.4. Affects Related Genes

Circadian genes play an important role in the functioning of the nervous system and memory. When circadian genes are severely disrupted by SD, memory function is affected [124]. According to a study, only 5 h of acute SD affected hippocampal gene expression [125]. A recent study found that up to 1146 genes were significantly dysregulated after 5 h of acute SD; these genes included 507 upregulated genes associated with RNA binding and processing and 639 downregulated genes associated with cell adhesion, dendritic localization, synapses, and postsynaptic membranes [126]. Further analysis of the GSE33302 and GSE9442 datasets revealed that seven circadian rhythm genes, including *Dicer1*, *Xbp1*, *Srebf1*, *Crem*, *Top1*, *Sfmbt1*, and *Naglu*, were significantly altered after SD; the SD models for both datasets exhibited reduced memory function [124]. In addition to regulating gene expression, SD may regulate transcription factors and RNA processing-related genes.

SD reduced the level of h3 and h4 acetylation of the BDNF gene promoter and impaired the function of key BDNF signaling pathways (pCaMKII, pErk2, and pCREB), resulting in a substantial reduction in BDNF expression [83]. Thus, BDNF and its protein acetylation regulation may play an important role in SD-induced effects on spatial memory. In addition, 24 h of REM SD decreased the expression of CREB and P-CREB in the DG region of the hippocampus [127]. Furthermore, 24 h of REM SD decreased the total CREB and P-CREB expression in the DG region of the hippocampus [82,128]. A serine/threonine kinase complex called mTORC1 regulates protein synthesis and influences synaptic plasticity and memory formation in the brain [129,130]. The activation of the PI3K/Akt/mTORC1 signaling pathway was found to be associated with improved neuroprotein synthesis, nerve regeneration, and neuronal survival [131]. In addition to reducing protein synthesis for 1 h, biochemical analyses indicated that SD weakened mTORC1 signaling in the hippocampus and that the total and phosphorylated mTOR levels and mTOR abundance were reduced by 5 h of SD [125]. In the mouse hippocampus, SD attenuated the phosphorylation of 4EBP2 by mTORC1 and the interaction between 4E and 4G [132]. Hence, SD could impair memory by attenuating mTORC1-dependent protein synthesis.

BMAL1 is the only gene capable of disrupting systemic rhythms. A deletion of BMAL1 in the forebrain impairs the functioning of the hippocampus. SD has a strong inhibitory effect on BMAL1 expression [133]. BMAL1 knockout mice have impaired hippocampus-dependent memory, as well as deficits in short- and long-term memory formation [134,135]. REM sleep-deprived rats showed impaired hippocampal working memory and concomitant decreases in the expression of the BMAL1 clock gene protein. The overexpression of Per1 prevents the decline in hippocampal memory associated with aging [136]. Per1 deficiency disrupts CREB-dependent gene expression and long-term hippocampal memory formation [136,137]. MT secretion and loss of rhythm in the clock genes mCry1 and mCry2 were observed in mice subjected to 3 days of SD. Homer1a is a scaffold protein that regulates synaptic plasticity and participates in learning and memory formation [138]. The Homer1a transcript levels were upregulated in the cortex and hippocampus after SD [139]. In transgenic mice, the overexpression of Homer1a impairs spatial working memory and long-term potentiation [140]. In transgenic mice, the overexpression of Homer1a impairs spatial working memory and long-term potentiation [141].

### 3.5. Neurotransmitter Changes

Neurotransmitter dysfunction is the main cause of SD, and neurotransmitters, such as amino acids, monoamines, cholinergic, and peptide neurotransmitters, are involved in sleep and affect memory function [142,143]. In the brain, several chemicals are involved in learning and memory, and current research on the effects of sleep disorders on learning and memory has primarily focused on changes in neurotransmitters, such as amino acids, monoamines, cholinergic compounds, and peptides.

#### 3.5.1. Amino Acids

SD may disrupt the release of amino acid neurotransmitters in the brain, resulting in a decrease in memory and learning abilities. Aminobutyric acid (GABA) plays a significant role in the advanced functions of the brain, such as memory and learning. Learning and memory abilities will be impaired as a result of the decrease in GABA levels. A number of studies have shown that the plasma concentrations of GABA and glutamic acid (Glu) are decreased in patients with insomnia [144,145]. In patients with primary insomnia, the average reduction in the brain GABA levels was 30%. The possible mechanism involves the regulation of the expression levels of GABA receptor subunit α-1 (GABA ARα1) and GABA acid receptor subunit γ2 (GABAARγ2) receptors in the hypothalamus and hippocampus [146]. After 24 h of REM SD, learning memory decreased and the Glu levels increased in the hippocampus of rats. This finding was significantly different from the levels of (152.10 ± 23.43) μmol·L^−1^ and GABA (422.50 ± 38.41) μmol·L^−1^ in control rats [53]. Memory storage was interfered by the Glu/GABA ratio [147]. The spatial learning and memory abilities of rats decreased significantly after 21 days of SD, similar to the ratio of Glu/GABA. As a consequence of SD-deprived glutamate and GABA concentration imbalance and reduced expression of glutamate receptors (NR2A and NR2B), synaptic transmission efficiency was reduced, leading to impaired memory performance [94]. Furthermore, the cognitive function of mice with reduced GAT-1 expression decreased [148]. The expression of GAT-1 in the brain stem and hippocampus of insomniac rats declined after 96 h of SD [149].

#### 3.5.2. Monoamines

The two types of monoamine neurotransmitters are catecholamines (dopamine, norepinephrine, melatonin) and indoleamines (5-HT). Increasing lines of evidence indicate that monoamine neurotransmitters play a significant role in the regulation of SD and memory function in the brain [9,150,151]. SD contributes to the decline of memory, as a study demonstrated that the serum NA level of medical staff as subjects decreased significantly [152]. Following different SD times (96, 120, 144 h), Wistar rats showed changes in monoamine neurotransmitters in the hypothalamus, with decreases in NA and DA and increases in 5-HT and 5-HIAA [153]. In rats, SD decreased the levels of NA in the brain, resulting in a reduction in their ability to learn and remember [154]. 5-HT regulates learning memory function and may be involved in the mechanism through which SD affects learning memory capacity. The 5-HT receptor subtype associated with memory is widely distributed throughout the central system. The plasma and brain 5-HT levels were significantly elevated in SD rats and increased with prolonged deprivation, resulting in central fatigue and reduced memory function [155]. The sleep–wake cycle is regulated by 5-HT and its receptors according to the literature [156,157]. In chronic SD rats, the 5-HT1a receptor mRNA transcript levels differed in the striatum, pontine reticular formation of the midbrain, and hypothalamus, with 5-HT concentrations being higher during wakefulness and lower during sleep [158]. In 18 healthy volunteers exposed to 24 h of SD, the levels of the 5-HT2a receptors were significantly increased in the ventral lateral prefrontal, parietal, and insulae [159].

Humans and animals require melatonin to regulate learning and memory processes [59,160,161]. A number of animal models and studies on the elderly have demonstrated that endogenous melatonin reduces memory deficits and modulates the retention of newly acquired information in long-term memory [162]. Moreover, melatonin reduces 5-fluorouracil-induced damage to hippocampal neurons and spatial memory in adult rats [160]. Short-term SD of 3 to 10 days can inhibit melatonin synthesis [138,163]. Similar findings were found for chronic SD, with C57BL/6J mice showing lower serum melatonin levels (2.33 ± 0.53) ng·mL^−1^ than controls (2.87 ± 0.23) ng·mL^−1^ after 10 consecutive weeks of incomplete SD intervention; moreover, the melatonin-mediated activity of the AMPK/SIRT1/PGC-1α energy metabolic pathway decreased [164]. According to correlation analysis, the CaMKII expression levels were positively correlated with learning and memory in rats. In the MT2/Ca^2+^/CaMKII/p-CREB pathway, melatonin-activated MT2 promotes increased intracellular calcium ions and the expression of CaMKII [165].

#### 3.5.3. Acetylcholine

Acetylcholine (Ach) plays an important role in learning and memory. The plasma levels of Ach were significantly reduced in patients suffering from insomnia and cerebral infarction [166]. In the brains of mice, 72 h of SD significantly reduced the amount of cholinergic neurotransmitter Ach [167]. Similarly, mice with 72 h of SD had reduced learning memory, reduced Ach content in the hippocampal tissue, and increased Ach E activity [168]. The hippocampal acetylcholine content and learning ability of rats were significantly reduced after 4 and 6 days of SD [169]. SD could impair the function of the hippocampal cholinergic system in mice, thereby impairing memory function.

#### 3.5.4. Peptides

Sleep–wake regulation is regulated by orexin, an excitatory neuropeptide produced by hypothalamic neurons, and activates two G-protein-coupled cell surface receptors, orexin 1 and orexin 2. By regulating ERK1/2, orexin-A promotes cell proliferation and is implicated in learning and memory [170]. The plasma orexin-A levels were found to be significantly higher in 228 patients with insomnia disorder than in normal sleepers (patients: 63.42 ± 37.56 pg·mL^−1^; normal; 54.84 ± 23.95 pg·mL^−1^) [171]. Furthermore, the orexin-A concentrations in the serum of 112 patients with primary insomnia were negatively correlated with their instantaneous and delayed memory scores [172]. Patients with AD and insomnia co-morbidities had higher peripheral blood orexin-A levels than the general population [173]. Researchers have suggested that impaired memory function in patients with insomnia is closely associated with elevated levels of orexin-A. Spatial learning memory and the proliferation of hippocampal dentate gyrus cells are influenced by orexin receptors [167]. The treatment of rats with 1 and 10 nmol·L^−1^ orexin-A impaired spatial memory [174]. The expression of OX1R and OX2R in the brain tissue of SD rats exposed to SD for 9 days increased after 6 h of SD [175]. The expression significantly increased in the hypothalamus [54,165]. In other cases, the orexin-A levels were significantly elevated in the cerebrospinal fluid of rats subjected to acute SD for 72 h [176]. Hence, SD may damage hippocampal neuronal cells by upregulating OX1R and OX2R expression and increasing the levels of orexin-A in the brain.

### 3.6. Circadian Rhythms

Circadian rhythm disorder can result in cognitive decline and impaired memory function [177]. Circadian rhythms are generated by molecular oscillators that interact with environmental and behavioral cycles to promote sleep during the night [178]. Studies have shown the significant role of circadian rhythms in regulating brain functions, including complex cognitive tasks [179,180,181,182]. Formation of the short-term and long-term memory is under strict circadian control [183]. Studies also suggest that common mechanisms underlie circadian rhythmicity and long-term memory formation [184]. The hippocampus shows circadian rhythmicity in pathways central to the memory-consolidation process [185]. Disrupting circadian rhythms impairs hippocampal memory in rats [180]. Additionally, hippocampal activity mediates the relationship between circadian activity rhythms and memory [186]. Cortical responses showed significant circadian rhythmicity, the phase of which varied across brain regions [187]. SD leads to memory impairment accompanied by changes in circadian rhythm-related genes [124]. The disorder of circadian genes affected by SD exacerbates the neuropathological damage of AD [188]. Studies have found that SD may modulate gliotransmission in the hypothalamus, thereby disturbing sleep–wake homeostasis and increasing susceptibility to neurological disease [189]. SD disrupts acquisition of contextual fear extinction by affecting circadian oscillation of hippocampal-infralimbic proBDNF [190].

### 3.7. Rodent-to-Human Complexities

The rodent-to-human translation is not straightforward. SD has been shown to impair memory in both rodent and human studies [18,191,192,193,194,195,196]. There remain many gaps in our understanding of brain development in rodents and humans, despite the fact that these are the species that have been most thoroughly evaluated to date [197]. While several rodent studies have investigated the impact of SD on memory, results are inconsistent [198]. Although some studies indicate that SD negatively affects memory, others have found no impairment and even improvements in memory performance [128,199]. For example, it was found that 24 h acute SD enhanced learning and memory in splenectomized rats [199]. This inconsistency has not been observed in human studies. The reason for these inconsistencies may be that although rodents have a similar brain structure to humans, their brains are significantly smaller, less complex, and less cognitively capable than humans [197,200,201,202,203]. Studies have found that the cortex in rodents is much less developed [202,204]. In summary, greater emphasis on human research and cross-species comparison is necessary to address the challenge of translating findings from rodent models to humans.

## 4. Summary and Prospects

This study examines the effects of SD on memory function in terms of synaptic plasticity, neuroinflammation, oxidative stress, genes, and neurotransmitters. This review contributes to a deeper understanding of the mechanisms underlying the effect of SD on memory.

SD alters the synaptic structure in the hippocampus, affecting synaptic plasticity as evidenced by altered BDNF levels and pCREB protein expression; it then disrupts long-term memory acquisition and consolidation. In addition, SD inhibits hippocampal neuronal regeneration, damages hippocampal neurons, and activates glial cells to release pro-inflammatory factors. This phenomenon increases neuritis, especially interleukin-like inflammatory factors, such as IL-6, which play a great role and should be considered in future studies. Oxidative stress contributes to memory impairment, which is accompanied by increased lipid oxidation. SD affects the expression of rhythmic and sleep genes, as well as modulates transcription factors and other mechanisms involved in memory function. Furthermore, SD impairs memory function by affecting the levels of relevant neurotransmitters.

A considerable uncertainty exists regarding the effects of SD on memory function. Currently, SD affects learning memory primarily through alterations in the levels of various endogenous substances, such as neurotransmitters. Future works should conduct in-depth investigations on pathways involved in sleep arousal and learning memory, which will be of significant clinical significance in discovering new targets to prevent SD and the resulting impairment of learning memory. SD contributes to memory impairment through a number of pathways, one or more of which is still unknown as the primary factor. Elucidating the exact mechanisms of SD-induced deficits will be a major research challenge. Future studies should examine the mechanisms by which SD impairs memory by examining the potential link between hippocampal neuroinflammation, oxidative stress, and kinase phosphorylation and cAMP response element-binding protein. Despite that sleep and memory are closely related to multiple brain regions, current research has concentrated on the hippocampus only. Future studies will focus on other relevant brain regions to investigate comprehensively and systematically the mechanisms underlying the effect of SD on memory. Furthermore, it is worth noting that the translation of rodent findings to humans requires a rigorous assessment of their validity and applicability, wherein the potential influence of circadian rhythmicity on experimental outcomes should be meticulously considered.

## Figures and Tables

**Table 1:** Memory function and SD: harmful effects.

Subject	Methods	SD Duration	Behavioral Tests	Impaired Memory	References
Humanity	Staying awake	25 h	Recognition test	Prospective memory Recognition memory	[13]
ICR mice	SIA	25 d	OLR, NOR, MWM	Short-term spatial and short-term nonspatial recognition memory; long-term spatial memory	[9]
C57BL/6 mice	Treadmill	3 d	MWM	Spatial memory	[51]
C57BL/6 mice	MMPM	72 h	OLR, NOR	Object position memory Object recognition memory	[52]
C57BL/6 mice	Gentle stimulation method	3 h	OPR	Long-term memory	[47]
3xTg-AD mice	MMPM	21 d	Y-maze Object identification	Recognition of memories Working memory Conditioned Fear Memory Y-maze memory	[32]
Mice	MMPM	72 h	Barnes maze task	Spatial learning and memory	[40]
SD rat	MMWP	7 d	Hexagonal Labyrinth Box	Recognition of memory	[53]
SD rat	Small platform–water environment	9 d	Small platform–water environment	Memory	[54]
SD rat	MMPM	7 d	MWM	Spatial memory	[55]
SD rat	Chronic and mild unpredictable stimulation	21 d	RAWM	Target quadrant memory	[56]
SD rat	MMPM	30 d	MWM	Memories of learning	[57]
SD rat	Automated cage-shaking apparatus	48 h	MWM	Spatial memory	[57]
SD rat	Gentle stimulation method	72 h	MWM	Spatial memory	[57]
SD rat	MMPM	72 h	Y-maze MWM	Spatial memory Recognition of memories Recognition of memories	[34]
SD rat	Gentle stimulation method	12 h	NOR, RAM	Spatial learning and memory	[35]
SD rat	Automatic TSD water box	24 h	The three-chamber paradigm test	Social interaction memory	[58]
Octodon degus	Automated device	24 h	MWM, NOR	Spatial learning and memory	[49]
Wistar rats	MMW	21 d	RAWM	Short- and long-term spatial memory	[37]
Wistar rats	MMPM	28 d	RAWM	Short- and long-term memory	[59]
Wistar rats	“Flower pot”	96 h	MWM	Memories of learning	[60]

OLR = object location recognition; NOR = novel object recognition; MWM = Morris water maze; SIA = automated sleep interruption apparatus; MMW = modified multi-platform–water environment; OPR = object place recognition.

## Data Availability

Not applicable.
